# Loop-mediated isothermal amplification (LAMP) assay for the diagnosis of fasciolosis in sheep and its application under field conditions

**DOI:** 10.1186/s13071-016-1355-2

**Published:** 2016-02-05

**Authors:** María Martínez-Valladares, Francisco Antonio Rojo-Vázquez

**Affiliations:** Faculty of Veterinary Medicine, University of León, Campus de Vegazana, 24071 León, Spain; Instituto de Ganadería de Montaña (CSIC-ULE), Finca de Marzanas, 24346 Grulleros, León Spain

**Keywords:** *Fasciola* spp, Sheep, Diagnosis, PCR, Loop-mediated isothermal amplification (LAMP)

## Abstract

**Background:**

Loop-mediated isothermal amplification (LAMP) is a very specific, efficient, and rapid gene amplification procedure in which the reaction can run at a constant temperature. In the current study we have developed a LAMP assay to improve the diagnosis of *Fasciola* spp. in the faeces of sheep.

**Findings:**

After the optimisation of the LAMP assay we have shown similar results between this technique and the standard PCR using the outer primers of the LAMP reaction. In both cases the limit of detection was 10 pg; also, the diagnosis of fasciolosis was confirmed during the first week post-infection in experimental infected sheep by both techniques. In eight naturally infected sheep, the infection with *F. hepatica* was confirmed in all animals before a treatment with triclabendazole and on day 30 post treatment in two sheep using the LAMP assay; however, when we carried out the standard PCR with the outer primers, the results before treatment were the same but on day 30 post-treatment the infection was only confirmed in one out of the two sheep. On the other hand, the standard PCR took around 3 h to obtain a result, comparing with 1 h and 10 min for the LAMP assay.

**Conclusions:**

The LAMP assay described here could be a good alternative to conventional diagnostic methods to detect *F. hepatica* in faeces since it solves the drawbacks of the standard PCR.

## Background

Species of *Fasciola* cause a zoonotic disease that is important from both economic and medical points of view in animals, principally ruminants. Its prevalence is increasing due to climate change, anthelmintic resistance [1] and man-made environmental modifications [2].

Diagnosis of fasciolosis is confirmed by the observation of parasite eggs in the faeces [3, 4] from seven weeks post-infection (wpi) onwards [5, 6]. Molecular methods were developed to increase the sensitivity and the specificity of conventional diagnosis, including immunological techniques [6]. However, PCR is only available to particular facilities because of the need for special detection devices. Another molecular technique called loop-mediated isothermal amplification (LAMP) is an alternative to PCR. LAMP assay is a very specific, efficient and rapid gene amplification procedure in which the reaction can run at a constant temperature [7]. In the current study we detected fasciolosis in experimentally and naturally infected sheep using the LAMP assay.

## Methods

Seven sheep were experimentally infected with 200 metacercariae of *Fasciola hepatica* each. Faecal samples were collected from rectum weekly from the day of infection until 8 wpi. Ethical approval was obtained from Insituto de Ganadería de Montaña ethics committee complying with national regulations (R.D. 53/2013). We also analysed by molecular methods the individual level of infection of 8 naturally infected sheep, previously tested by Robles-Pérez et al. [8], before and after 30 days post-treatment (dpt) with triclabendazole. The number of *F. hepatica* eggs was calculated by a sedimentation method.

At least 1 g faeces per sheep was collected weekly for a pool of faeces of all experimentally infected animals. DNA extraction was applied to 0.5 g of each weekly pool using “SpeedTools Tissue DNA Extraction Kit” (Biotools, Madrid, Spain. Regarding natural infection, DNA samples from faeces collected in the study by Robles-Pérez et al. [8] were analysed.

DNA from adult worms was also extracted as previously is indicated in faeces. Serial 10-fold dilutions from *F. hepatica* DNA were prepared ranging from 10^−1^ ng/μl to 10^−6^ ng/μl to determine the sensitivity. A sample with 10^−1^ ng/μl DNA was used as a positive control in all reactions. DNA from other helminths (*Dicrocoelium dendriticum*, *Calicophoron daubneyi*, *Teladorsagia circumcincta*, *Haemonchus contortus* and *Trichostrongylus colubriformis*) was also extracted to determine the specificity.

**Fig. 1:**
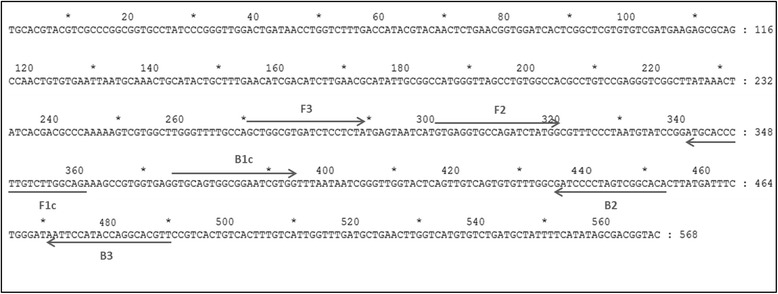
LAMP primer set targeting the consensus sequence of the internal transcribed spacer 2 (ITS2) region of *Fasciola* spp

Primers used for the LAMP assay were designed based on a highly conserved region of *Fasciola* spp. genome. Genbank sequences (http://blast.ncbi.nlm.nih.gov/ Blast.cgi) (Table 1) including the internal transcribed spacer 2 (ITS2) region were tested *in silico* through BLAST searches and alignment analysis using the ClustalW module of the MegAlign software (DNASTAR Inc., Madison, WI, USA). A 568 base pairs (bp) consensus sequence was selected for the design of specific primers using Primer Explorer V4 (http://primerexplorer.jp/e/). Two sets of primers comprising two outer (F3 and B3) and two inner (FIP and BIP) were selected. FIP contained the F1c (complementary to F1) and F2 sequences. BIP contained the B1c (complementary to B1) and B2 sequences (Fig. 1 and Table 2).

**Table 1:** GenBank accession numbers for the different *Fasciola* spp. isolates included in the alignment

Isolates	GenBank accession number
*Fasciola* sp.	JF708043
*F. hepatica*	GQ231547
*F. hepatica*	JF708026
*F. hepatica*	JF708036
*F. hepatica*	HM746786
*F. hepatica*	AM709622
*F. hepatica*	JF432071
*F. gigantica*	JF432074
*F. gigantica*	JF496714
*F. gigantica*	KF425321
*F. gigantica*	AM850108
*F. gigantica*	HM746788
*F. gigantica*	JN828956

**Table 2:** LAMP primer sequences. F3, forward outer primer; B3, reverse outer primer; FIP, forward inner primer (comprising F1c and F2 sequences); BIP, reverse inner primer (comprising B1c and B2 sequences)

Primers	Sequence (5’-3’)
F3	GCTGGCGTGATCTCCTCTA
B3	AACGTGCCTGGTATGGAATT
FIP (F1c-F2)	TCTGCCAAGACAAGGGTGCAT-GTGAGGTGCCAGATCTATGG
BIP (B1c-B2)	GTGCAGTGGCGGAATCGTGG-TGTGCCGACTAGGGGATC

A standard PCR, using 0.5 μM of primers F3 and B3 (Table 2) and 1 μl (20 ng/ μl) DNA, was carried out in a total volume of 20 μl following previously published protocol [6] with annealing temperature of 63 °C for 30 s. Amplification products were analysed by electrophoresis in 2 % agarose TBE (Tris base, boric acid and EDTA). All reactions were carried out in triplicate. One of the resulting bands was sequenced at the Laboratorio de Técnicas Instrumentales (León, Spain).

LAMP assay was set up testing different betaine, MgSO_4_ concentrations and temperatures. LAMP reaction mixtures (25 μl) contained 40 pmol of each FIP and BIP primers, 5 pmol of each F3 and B3 primers, 1.4 mM of each dNTPs (Biotools, Spain), 1× Isothermal Amplification Buffer (New England Biolabs, Beverly, MA, USA), betaine (0.8, 1, 1.2, 1.4, or 1.6 M) (Sigma, city, USA), MgSO_4_ (4, 6, 8, or 10 mM) (New England Biolabs, UK) and 8U of Bst 2.0 WarmStart DNA polymerase (New England Biolabs, UK), with 1 μl of DNA (20 ng/ μl). Different temperatures were tested using a thermocycler (Bio-Rad) set at 61, 63, and 65 °C for 60 min and then at 80 °C for 10 min. Amplifications were visually detected by adding 2 μl of 1:10 diluted 10,000× concentration fluorescent dye SYBR Green I (Invitrogen, Carlsbad, CA USA). Green fluorescence was observed in successful LAMP reactions; it remained orange in the negative reactions. The LAMP reaction products were also analysed by electrophoresis in 2 % agarose TBE. All reactions were done in triplicate.

## Results

**Fig. 2:**
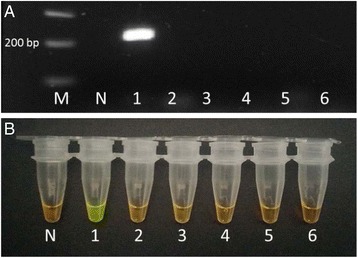
Specificity of standard PCR with the primers F3 and B3 of the LAMP assay (**a**) and LAMP assay (**b**). M: marker. N: negative control. DNA samples from *Fasciola hepatica* (1), *Dicrocoelium dendriticum* (2), *Calicophoron daubneyi* (3), *Teladorsagia circumcincta* (4), *Haemonchus contortus* (5) and *Trichostrongylus colubriformis* (6)

*In silico* comparisons of LAMP primers showed a homology of 100 % with *F. hepatica* and *F. gigantica*. The specificity of the outer primers, F3 and B3, was carried out by means of a standard PCR and LAMP reaction (Fig. 2). Primers amplified a band of 220 bp; after sequencing we showed a 100 % homology with the sequence shown in Fig. 1, consensus sequence of the ITS2 region of *Fasciola* spp . The optimisation of the LAMP reaction was done with 1 M betaine, 8 mM MgSO_4_ and incubation at 63 °C.

**Fig. 3:**
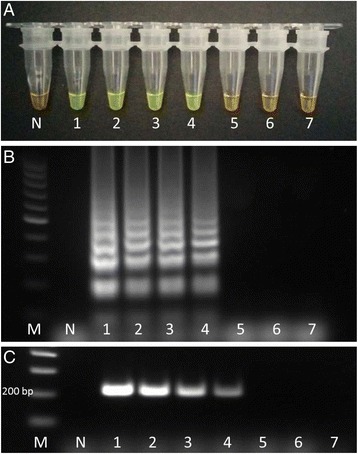
LAMP assay, 10-fold serial dilution of DNA from *F. hepatica*. **a** LAMP reaction after adding SYBR Green I; **b** LAMP reaction products analysed by electrophoresis in 2 % agarose gel; **c** Standard PCR with the primers F3 and B3 of the LAMP assay. N: negative control; 1–7: 10-fold serial dilution of DNA from *F. hepatica* adult worm ranging from 1 ng/ml (1) to 10^−6^ ng/ml (7); M: marker

The limit of detection of LAMP reaction was 10^−3^ ng/μl (Fig. 3a); the amplification products were visualised on an agarose gel as a ladder of multiple bands (Fig. 3b). The sensitivity of the standard PCR with the primers F3 and B3 was also 10^−3^ ng/μl (Fig. 3c).

**Fig. 4:**
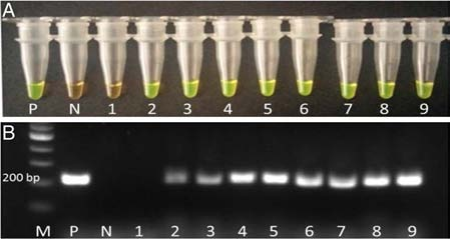
Detection of the infection in experimentally infected sheep. **a** LAMP reaction after adding SYBR Green I; **b** Standard PCR with the primers F3 and B3 of the LAMP assay. P: positive control; N: negative control; 1–9: pooled samples of sheep on day 0 of the infection (1) and between 1 and 8 weeks post infection (2–9); M: marker

In relation to the experimental infection, the first egg excretion was detected at 8 wpi and only in two sheep with a mean of 30 eggs per gram (epg); at 10 wpi the infection was confirmed in all animals. Using the LAMP assay and the standard PCR, the infection was detected from the first wpi onwards (Fig. 4a, b respectively).

**Fig. 5:**
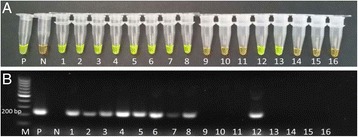
Detection of the infection in naturally infected sheep. **a** LAMP reaction after adding SYBR Green I; **b** Standard PCR with the primers F3 and B3 of the LAMP assay. P: positive control; N: negative control; 1–8: individual samples of infected sheep on day 0 of treatment; 9–16: individual samples of infected sheep on day 30 post-treatment; M: marker

In naturally infected sheep, all animals were infected and the mean epg was 84.4 ± 58.3 on day 0 of the experiment; however, on 30 dpt all sheep had a value of 0 epg [8]. Using the LAMP assay the infection was confirmed in all animals before the treatment although at 30 dpt two sheep resulted positive (Fig. 5a). With the standard PCR, we also found all positive sheep on day 0 although after treatment we only confirmed the infection in one out of the two sheep diagnosed by the LAMP assay (Fig. 5b).

## Discussion

Our aim was to develop a qualitative LAMP assay for the detection of *F. hepatica* in faeces, with the aim to improve certain properties associated with PCR, such as being a time-consuming method that requires complex devices. In the current study, the standard PCR took around 3 h to obtain a result, comparing with 1 h and 10 min with the LAMP assay. On the other hand, the advantage of this qualitative method is that the reaction can be carried out on a block heater and it allows an easy visualisation of the result using fluorescence. Quantitative LAMP assays have also been described although expensive devices are needed. In these assays the reaction was translated on to a real-time PCR platform [9] or results were monitored measuring the turbidity through spectrophotometric analysis [10].

This LAMP assay was designed to detect the infection by any species of *Fasciola* in faeces, although we only have tested it in sheep infected with *F. hepatica*.

The sensitivity of the LAMP assay and the standard PCR was 1 × 10^−3^ ng or 10 pg. Similar results were described by Li et al. [10], who reported a limit of detection of 0.3 × 10^−3^ ng for a LAMP assay to detect *Trichinella spiralis* and 3.6 × 10^−3^ ng by means of a standard PCR using also the outer primers. However, Melville et al. [9] showed that the sensitivity to detect the infection by *H. contortus* in faecal samples from sheep was 10^−5^ ng/μl. Ai et al. [11] carried out a species specific LAMP assay to discriminate between *F. hepatica* and *F. gigantica* showing a limit of detection of 10^−5^ ng; these primers could be tested in faecal samples to distinguish between species in epidemiological studies.

In experimentally infected sheep, we were able to diagnose the infection from the first week pi onward with both techniques, the LAMP assay and the standard PCR. The DNA detected at this stage of the infection could proceed from cellular material from tegumental turnover and repair during host's immune response [12] or maybe from metacercariae that did not progress. Previously, the detection of *F. hepatica* was possible from two wpi using a nested and another standard PCR with DNA from faeces [8, 12].

We tested the LAMP assay with DNA samples of naturally infected sheep previously analysed by Robles-Pérez et al. [8]. We found that all animals were infected by *F. hepatica* using the LAMP assay and the standard PCR, in accordance with the result obtained by these authors, who used a different pair of primers to amplify a region of the ITS2. In samples collected at 30 dpt, we detected the infection in two out of eight sheep using the LAMP assay, although only in one of these two sheep using the standard PCR. However, Robles-Pérez et al. [8] did not find any positive sheep after treatment using PCR, sandwich ELISA, or direct visualisation of eggs in faeces. Occasionally, fluke eggs can be detected in faeces as false positives due to their presence in the gall-bladder some days after successful treatment [13]. Accordingly, the molecular techniques could amplify DNA from these residual eggs. The positive animals at 30 dpt suggest that the sensitivity of the LAMP assay is slightly higher than the standard PCR; however, these results do not confirm the resistance to triclabendazole since the slaughter of sheep to determine its efficacy was not possible.
